# Long-term outcomes of co-administration of CD19 and CD22 CAR-T cell therapy in pediatric patients with relapsed/refractory Philadelphia chromosome-positive acute lymphoblastic leukemia

**DOI:** 10.3389/fmed.2026.1815353

**Published:** 2026-04-10

**Authors:** Zichao Zhou, Tianyi Wang, Wenhua Shi, Xinyu Wan, Wenjie Li, Yue Li, Jing Yang, Xiaomin Yang, Liu Yang, Jing Zhang, Meng Su, Kang An, Xiang Wang, Hua Zhu, Chengjuan Luo, Jun Lu, Jing Chen, Lili Song, Yanjing Tang, Benshang Li

**Affiliations:** 1 Department of Hematology/Oncology, National Health Committee Key Laboratory of Pediatric Hematology & Oncology, Shanghai Children’s Medical Center, Shanghai Jiao Tong University School of Medicine, Shanghai, China; 2 Department of Hematology/Oncology, Children’s Hospital of Soochow University, Suzhou, Jiangsu, China

**Keywords:** allo-HSCT, CAR-T cell therapy, CNS, Ph^+^ ALL, relapsed or refractory

## Abstract

**Background:**

Despite initial responses, resistance to tyrosine kinase inhibitors (TKIs) and disease relapse remain major challenges in patients with Philadelphia chromosome-positive acute lymphoblastic leukemia (Ph^+^ ALL). Given the efficacy of chimeric antigen receptor T (CAR-T) cell therapy in Relapsed/Refractory (R/R) ALL, we aimed to evaluate the long-term outcomes of co-administration of CD19 and CD22 CAR-T cell therapy for pediatric patients with R/R Ph^+^ ALL.

**Methods:**

We conducted a retrospective subanalysis of patients with R/R Ph^+^ ALL to assess the long-term outcomes of co-administration of CD19 and CD22 CAR-T cell therapy. Complete hematologic remission (CHR), measurable residual disease-negative complete remission (MRD^−^CR), complete molecular remission (CMR), relapse-free survival (RFS), and overall survival (OS) were among the important outcomes that were evaluated with a data cutoff of October 1, 2025.

**Results:**

Within 1 month after CAR-T cell infusion, all patients achieved CHR, with MRD^−^CR and CMR of 100 and 77.8%, respectively. Two patients underwent consolidative allogeneic stem cell transplant (allo-HSCT). Notably, six patients achieved sustained CHR without allo-HSCT. At a median follow-up of 54.93 months (range, 16.83–71.6 months), the 4-year OS and RFS were 91.7 and 66.7%. No treatment-related deaths occurred from CAR-T toxicity.

**Conclusion:**

These preliminary findings suggest that CD19 and CD22 CAR-T cell therapy may provide long-term survival benefits in pediatric patients with R/R Ph^+^ ALL, with manageable toxicity. However, these results should be considered hypothesis-generating and require validation in larger, controlled studies.

**Clinical Trial Registration:**

https://www.chictr.org.cn/showproj.html?proj=52403, ChiCTR2000032211.

## Introduction

Acute lymphoblastic leukemia (ALL) is the most common childhood leukemia, with Philadelphia chromosome-positive (Ph^+^) ALL representing 3–5% of cases and historically associated with poor outcomes and high relapse rates, including frequent central nervous system (CNS) involvement (1, 2). This subtype is characterized by the t(9;22)(q34;q11) chromosomal translocation, which generates the *BCR::ABL1* fusion protein, most commonly in the p190 or p210 isoforms. The prognosis of conventional chemotherapy fell short of expectations, with 3-year overall survival (OS) of only 5–15% (3, 4). Although the addition of the tyrosine kinase inhibitors (TKIs) has considerably improved outcomes, relapse after initial treatment and TKI resistance are still a critical issue that needs to be resolved (5–7). Consequently, modern management focuses on mutation-guided TKI selection combined with novel immunotherapies (8).

Chimeric antigen receptor T (CAR-T) cell therapy targeting CD19 has transformed the treatment landscape for Relapsed/Refractory (R/R) ALL in both children and adults (9, 10). A recent study of 56 patients with R/R Ph^+^ ALL showed that CD19-targeted CAR-T therapy was highly effective, with a 91.4% complete remission (CR) rate resulting in a 67.9% rate of minimal residual disease (MRD)-negative status (11). Beyond its efficacy in bone marrow relapse, CAR-T therapy has demonstrated promising activity against CNS leukemia (CNSL), including in Ph^+^ ALL (12). However, approximately 30–50% of R/R ALL patients still relapse after CD19-targeted CAR-T therapy due to loss of CD19 antigen or lineage switch (10, 13). CD22 represents another important marker on leukemic blasts, and CD22-targeted CAR-T cell therapy could induce complete remission in 70–80% (14). Similarly, the CD3/CD19 bispecific T-cell engager blinatumomab and the CD22- targeted antibody- drug conjugate inotuzumab ozogamicin have demonstrated clinical activity in R/R Ph^+^ ALL. However, the efficacy of blinatumomab appears limited in patients with high disease burden or extramedullary disease (EMD), and the long-term outcomes of both agents as monotherapies remain suboptimal (9, 15, 16).

To address these limitations, our study employed co-administration of CD19 and CD22 CAR-T cells to reduce the risk of antigen-negative relapse and improve durability. Furthermore, the long-term outcomes of CD19 and CD22 dual-target CAR-T therapy in pediatric patients with R/R Ph^+^ ALL remain incompletely characterized.

Here, we report long-term outcomes of co-administration of CD19 and CD22 CAR-T cell therapy for pediatric patients with R/R Ph^+^ ALL, retrospectively analyzing clinical characteristics, treatment responses, safety, and CAR-T cell persistence.

## Methods

### Study design and treatment

**Figure 1:**
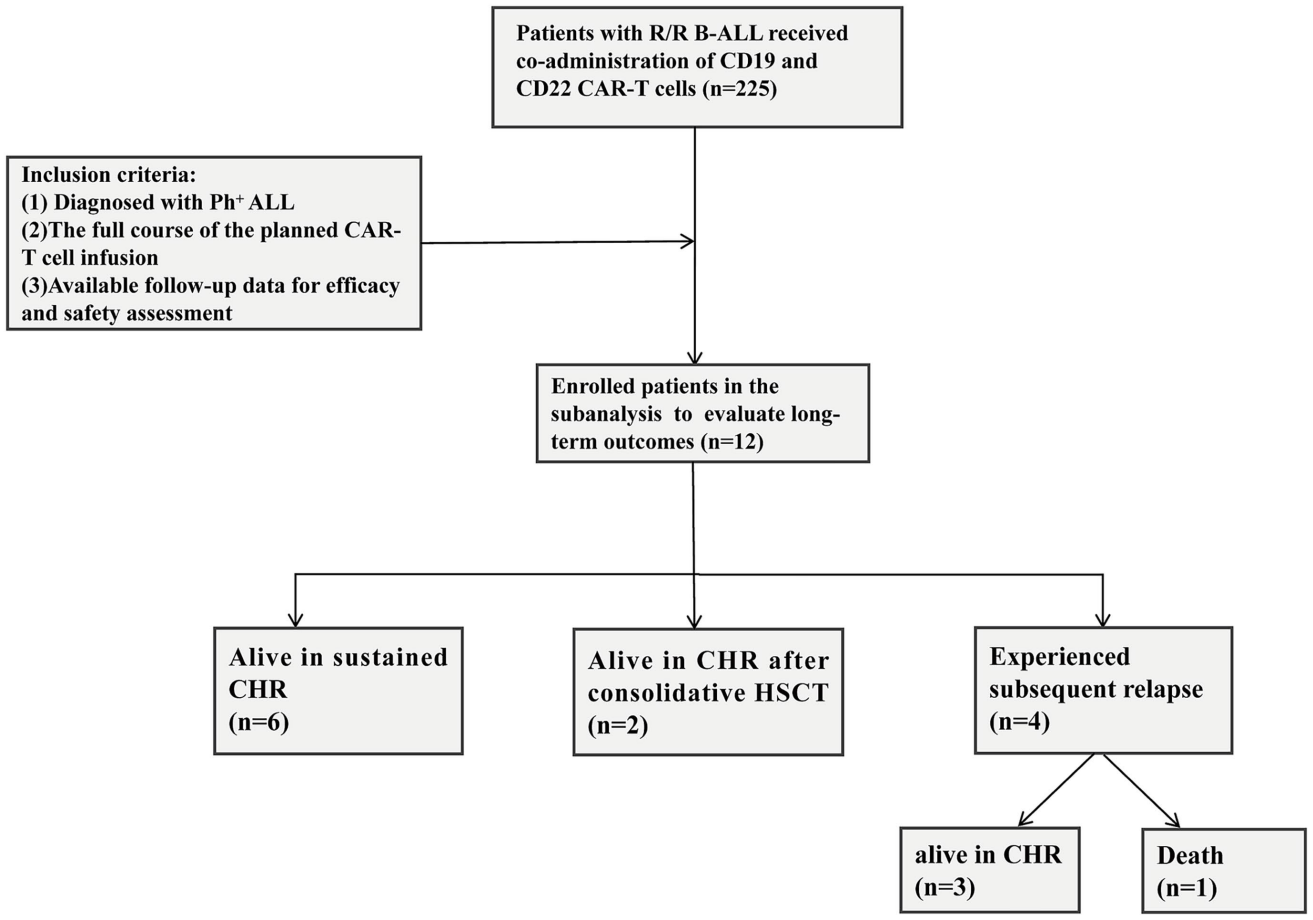
Consort diagram.

The current study is a retrospective subanalysis based on a prospectively maintained database derived from the parent phase II trial (Trial registration: ChiCTR2000032211). The parent phase II trial enrolled 225 pediatric patients with R/R B-ALL between September 17, 2019, and December 31, 2021. Definitions of R/R disease status were based on the National Comprehensive Cancer Network (NCCN) Guidelines for ALL. The inclusion criteria for the present cohort were defined retrospectively, rather than as a predefined subgroup analysis of R/R Ph^+^ ALL patients within the original trial protocol (17). Specifically, all patients enrolled in the parent trial who met the following criteria were consecutively included in this subanalysis: (1) diagnosed with Ph^+^ ALL; (2) received the full course of the planned CAR-T cell infusion; and (3) had available follow-up data for efficacy and safety assessment. No additional exclusion criteria were applied beyond those of the parent trial (17) (Figure 1). Diagnosis of Ph^+^ ALL was confirmed by fluorescence *in situ* hybridization (FISH) or real-time quantitative polymerase chain reaction (qPCR) according to the World Health Organization classification.

This subanalysis was approved by the ethics committee of Shanghai Children’s Medical Center, and informed consent was obtained from each participant, in compliance with the Declaration of Helsinki. The data cutoff date was October 1, 2025. Data were retrospectively collected by reviewing electronic medical records. Additional follow-up information was obtained from family members when direct clinical documentation was unavailable.

All enrolled patients underwent lymphodepleting chemotherapy with cyclophosphamide (500 mg/m^2^ × 2 days) and fludarabine (40 mg/m^2^ × 3 days) on days - 4 to - 2 and received a single intravenous infusion of dual CD19 and CD22 CAR-T cells on day 0 (Supplementary Figure S1). Prior to lymphodepletion, leukemia immunophenotyping was performed by Flow Cytometry (FCM) to confirm CD19 and CD22 target expression. Tumor burden assessments were performed for bone marrow (BM) and cerebrospinal fluid (CSF) before CAR-T infusion. Specifically, BM was examined for cytomorphology and measurable residual disease (MRD) according to the guidelines of Shanghai Children’s Medical Center, with BM morphology classified as M1 (<5% blasts), M2 (5–25% blasts), or M3 (>25% blasts). In patients with central nervous system (CNS) involvement, CSF samples were collected via lumbar puncture and analyzed cytologically to assess CNS status.

### CAR-T cells manufacturing

The CD19 and CD22 CAR constructs contained the FMC63 and the m5/44 single chain variable fragment (scFv) domains, the CD8α hinge and transmembrane region, and the 4-1BB costimulatory and CD3-zeta signaling domains, respectively (Supplementary Figure S1). Peripheral blood mononuclear cells (PBMC) were isolated from the PB of patients, and T cells were separated and stimulated with anti-CD3/CD28 Dynabeads™ (Gibco, Lithuania). After 2 days of activation, T cells were separately transduced with a GMP lentiviral vector encoding the CD19- and CD22-targeted CAR. Transduction efficiency was assessed 2 days later. The CD19-targeted CAR-T cells and CD22-targeted CAR-T cells were then expanded separately in TexMACS GMP Medium (Miltenyi, Germany) supplemented with IL-7 and IL-15 (Miltenyi, Germany) for 7 to 10 days, resulting in two separate autologous CAR-T cell products. For administration, the two products were mixed in a single infusion bag and administered together as a combined infusion. The proportion of the two components was nearly fixed as a 1:1 ratio based on the number of viable CAR-positive T cells (Supplementary Table S1).

Regarding manufacturing and release criteria, all participating centers followed a harmonized protocol, with central testing for key release parameters to ensure consistency. The manufacturing processes were identical across centers, and release criteria were uniform, including viability (>70% examined by Trypan Blue), transduction efficiency (>20% examined by FCM).

### Clinical response assessments

Complete hematologic remission (CHR) was defined as <5% BM blasts and no lymphoblasts in the CSF. MRD-negative CR (MRD^−^CR) was defined as <0.01% leukemia cells detected in BM or CSF by 8-color BD FACS Canto II FCM (San Jose, CA, United States) with the following antibody panel: CD10-PECy7/CD34-PerCP/CD22-APC/CD19-APCH7/ CD20-V450/CD45-V500 (BD, San Jose, CA, United States).

Complete molecular remission (CMR) was defined as the absence of detectable *BCR::ABL1* transcripts (*BCR::ABL1*/ABL1 < 0.01%) in bone marrow samples. Molecular response was assessed by quantifying *BCR::ABL1* transcript levels using qPCR with a sensitivity of 1 × 10^−4^ in bone marrow samples. ABL1 kinase domain mutations and other relevant genomic alterations were analyzed by RNA sequencing at the time of diagnosis or relapse. Relapse was defined as the reappearance of blasts in the BM, peripheral blood (PB), and/or the presence of extramedullary disease after achieving CHR.

FCM was also used to assess the CAR-T cells persistence in PB, BM, and CSF by quantifying the percentage and absolute count of CAR-T cell and CD3^−^ CD19^+^ B cells. CAR gene copies per μg DNA were measured by qPCR and normalized to the single-copy gene, CDKN1a. B-cell aplasia (BCA) was defined as meeting either of the following criteria in PB or BM: (1) a <1% proportion of CD3^−^19^+^ B cells in white blood cells, or (2) an absolute B-cell count ≤ 200/μL among lymphoblasts.

The BD™ Cytometric Bead Array (CBA) Human Th1/Th2/Th17 Cytokine Kit (BD, San Jose, CA, United States) was utilized to measure serum concentrations of cytokines, including interleukin (IL)-2, IL-4, IL-6, IL-10, IL-17A, tumor necrosis factor-α (TNF-α), and interferon-γ (IFN-γ). Cytokine release syndrome (CRS) and immune effector cell-associated neurotoxicity syndrome (ICANS) were graded according to American Society for Transplantation and Cellular Therapy criteria (ASTCT) (18). Other adverse events (AEs), including hemocytopenia and infection, were graded according to the NCI Common Terminology Criteria for Adverse Events (CTCAE), version 5.0.

### Statistical analysis

Descriptive statistics were used to summarize patient characteristics, AEs, and efficacy. Relapse-free survival (RFS) was defined as the time from CAR-T cell infusion to the first documented relapse or death from any cause. Overall survival (OS) was defined as the time from CAR-T cell infusion to death from any cause, censored at the date of last follow-up. RFS and OS were estimated using Kaplan–Meier curves. The differences between groups were examined using 2-sided log-rank tests. Continuous variables were determined with the Mann–Whitney test, and categorical variables were determined with a Fisher’s two-sided exact test for two-group comparisons. OS, RFS, and CMR with 95% CI were calculated using the Clopper-Pearson method. Statistical analyses were performed using GraphPad Prism 9 and SPSS Statistics 27 software. A two-tailed *p* value threshold of < 0.05 was set for statistical significance.

## Results

### Patient baseline and CAR-T cell therapy characteristics

A total of 12 R/R Ph^+^ acute lymphoblastic leukemia patients were enrolled and successfully received co-administration of CD19 and CD22 CAR-T cell infusion. Three (25%) patients had been exposed to ≥2 TKIs. Of these patients, 5 (41.7%) had BM involvement, 4 (33.3%) had combined BM and CNS involvement, and 3 (25%) had isolated CNS relapse. The median age was 10.42 years (range, 5.67–17.00 years). Among them, 8 (66.7%) patients had p190 Ph^+^ ALL, while 4 (33.3%) had p210 Ph^+^ ALL. ABL1 kinase domain mutations were present in 1 (8.33%) patient, and 5 (41.7%) patients had other genetic variations. Before CAR-T cell infusion, bone marrow blasts of ≥5% were found in 5 (41.7%) patients, and 4 (33.3%) had CNS-3 disease status (Table 1; Supplementary Table S2).

**Table 1:** Patient characteristics and clinical outcome.

Patient ID	Gender/age	Disease status	Tissue involvement	Transcript at diagnosis	Genetic variation	Marrow disease burden	Treatment lines	Prior TKIs	^+^Response to CAR-T	RFS (M)	OS (M)	Last status
Pt01	F/8.77	Relapse	^*^CNS-2	p190	/	MRD^neg^	2	Dasatinib	CHR	7.40	52.70	Alive
Pt02	M/15.00	Relapse	BM, ^*^CNS-3	p190	/	M2	2	Dasatinib	CHR	9.13	50.27	Alive
Pt03	F/6.26	Refractory	BM	p210	ABL1: T315I, E225K	M2	1	Imatinib; Dasatinib; Ponatinib	CHR	71.60	71.60	Alive
Pt04	F/9.03	Relapse	BM	p210	WHSC1: E1099K; KMT2D: Q2731X; IKZF1 del	M3	2	Dasatinib	CHR	11.73	52.30	Alive
Pt05	M/11.68	Relapse	BM, ^*^CNS-1	p190	IKZF1 del	M1	2	Imatinib; Dasatinib	CHR	48.77	48.77	Alive
Pt06	M/10.92	Relapse	BM, ^*^CNS-3	p190	/	M2	4	Dasatinib	CHR	58.93	58.93	Alive
Pt07	M/17.00	Relapse	BM	p190	IKZF1-IK6	M1	2	Dasatinib	CHR	2.43	16.83	Died
Pt08	M/5.69	Relapse	^*^CNS-3	p190	BCORL1:pTrp1622fs	MRD^neg^	2	Dasatinib	CHR	52.27	52.27	Alive
Pt09	M/10.82	Relapse	^*^CNS-3	p190	/	MRD^neg^	2	Dasatinib	CHR	57.17	57.17	Alive
Pt10	M/12.26	Relapse	BM	p190	/	M1	2	Dasatinib	CHR	57.97	57.97	Alive
Pt11	M/10.02	Relapse	^*^CNS-1	p210	/	MRD^neg^	2	Dasatinib	CHR	60.00	60.00	Alive
Pt12	M/9.78	Relapse	BM	p210	/	M3	2	Imatinib; Dasatinib	CHR	60.67	60.67	Alive

F, Female; M, Male; pt01, patient 01; /, None.

Bone marrow (BM) blasts by morphology: M1 (<5% blasts), M2 (5–25% blasts), M3 (>25% blasts); MRD: minimal residual disease, MRD^neg^: MRD < 0.01% leukemia cells; ^+^Response to CAR-T: disease assessments for BM and CSF on Day 28 after CAR-T cell infusion.

^*^CNS: central nervous system; CNS-1: absence of lymphoblasts in the cerebrospinal fluid (CSF), regardless of the white blood cells (WBCs) count; CNS-2: WBCs count <5/μL, with the presence of lymphoblasts in the CSF; CNS-3: WBCs count >5/μL, with concurrent detection of lymphoblasts in the CSF.

CHR, complete hematologic remission; NA, not available; TKIs, tyrosine kinase inhibitors; RFS, Relapse-free survival; OS, overall survival.

The median total dose of CD19 and CD22 CAR-T cells was 4.57 × 10^6^/kg (range, 2.3 × 10^6^ − 1.12 × 10^7^/kg). The median doses of CD19 CAR-T cells and CD22 CAR-T cells were 2.35 × 10^6^/kg (range, 0.8 × 10^6^ − 5.6 × 10^6^/kg) and 2.25 × 10^6^/kg (range, 1.2 × 10^6^ − 6.3 × 10^6^/kg), respectively. The median CAR transduction efficiencies were 44.1% for CD19 CAR-T cells (range, 26.8–74.0%) and 44.65% for CD22 CAR-T cells (range, 24.9–77.4%) (Supplementary Table S1).

### Efficacy and long-term survival

Within 1 month after CD19 and CD22 CAR-T cell infusion, all patients achieved CHR, with the MRD^−^CR of 100% (95% CI, 73.5–100%). Separately, one patient (Pt03) underwent consolidative allogeneic stem cell transplant (allo-HSCT) after 2 months of CAR-T therapy and was still alive with CMR for over 70 months. Except for three patients lacking molecular data, seven of nine patients (77.8, 95% CI, 40–97.2%) achieved CMR (Supplementary Table S3). Of the two who did not, Patient 12 (Pt12) attained CMR after treatment with dasatinib, ponatinib, and consolidative allo-HSCT, while Pt02 experienced relapse at 9 months.

**Figure 2:**
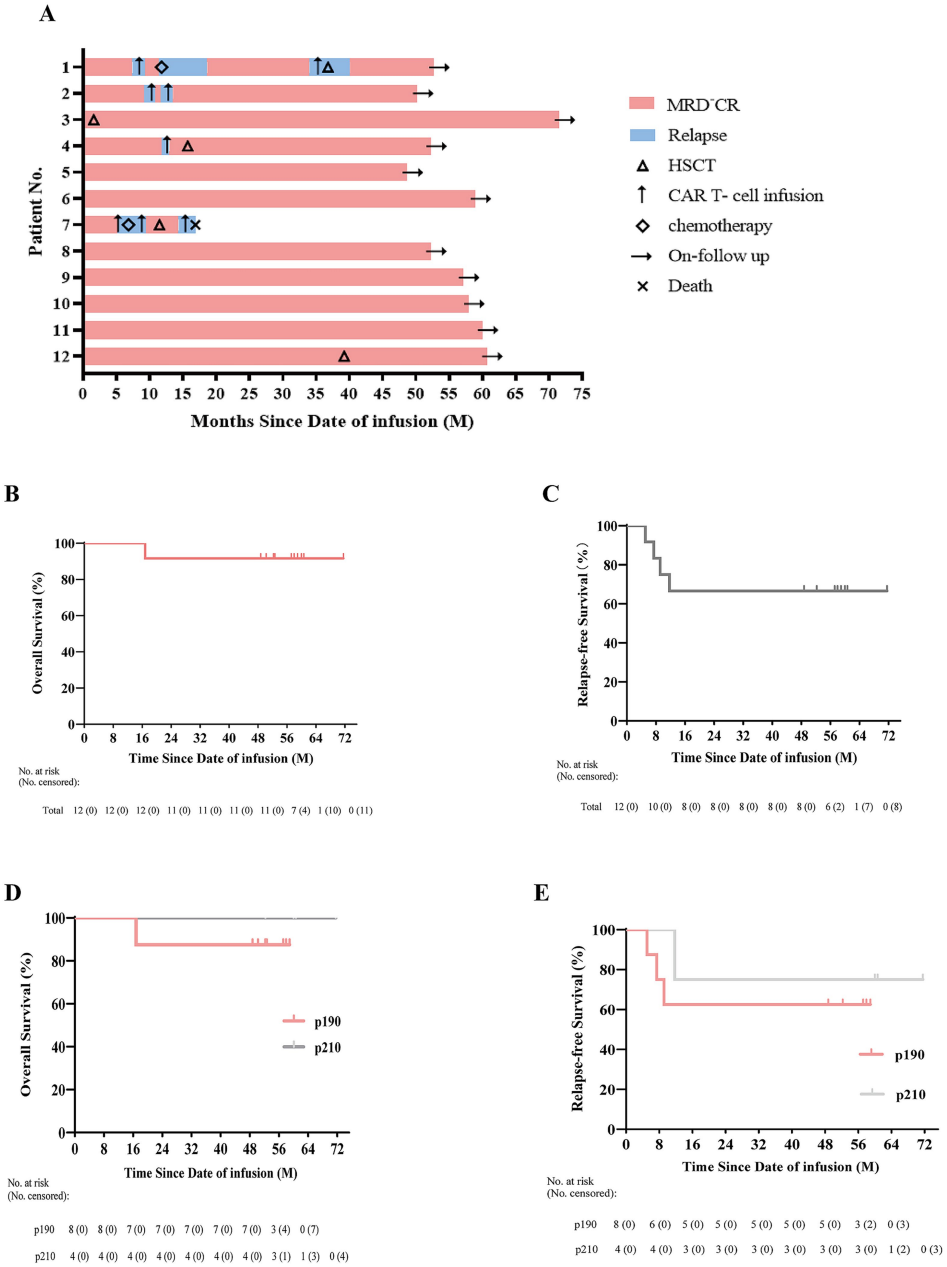
Clinical courses after CAR-T cell therapy. **(A)** Swimmer plot illustrating the clinical response and follow-up status of individual Ph^+^ ALL patients (*n* = 12). The color of each bar corresponds to different response statuses, while the length of each bar represents a patient’s survival duration, calculated from the date of CAR-T cell infusion to either the date of death or the last follow-up. **(B,C)** Kaplan–Meier curves of overall survival (OS) and relapse-free survival (RFS) of 12 R/R Ph^+^ patients. **(D,E)** Overall survival and relapse-free survival according to the fusion protein types p190 (*n* = 8) and p210 (*n* = 4). Tick marks indicate the time of censoring. No. at risk = number at risk; No. censored = number censored.

Four patients (33.3%) experienced CD19^+^/CD22^+^ relapse, and no patient developed CD19-negative relapse. All of them received secondary CD19/CD22 CAR-T cell therapy and three patients regained MRD^−^CR. Among them, one patient (Pt04) received allo-HSCT consolidation and remains alive. However, three other patients experienced two or three relapses after subsequent CAR-T cell therapy, requiring a combination of CAR-T cell, TKIs, and HSCT therapy (Supplementary Table S4). Two of three patients (Pt01, Pt02) regained CHR and are still alive. Unfortunately, Pt07 died from septic shock after multiple relapses. Figure 2A presents an overview of the 12 patients’ clinical courses.

At a median follow-up of 54.93 months (range, 16.83–71.6 months), the 4-year OS was 91.7% (95% CI, 61.5–99.8%) and the 4-year RFS was 66.7% (95% CI, 34.9–90.1%), respectively (Figures 2B,C). *BCR::ABL1* positivity was detected in five (41.7%, 5/12) patients after CAR-T cell therapy, two of whom (Pt02, Pt07) experienced hematological relapse. At the cutoff date, eleven (91.7%) patients remained in CHR. Notably, six patients achieved sustained CHR without allo-HSCT.

Given the small sample size, definitive conclusions cannot be drawn; however, patients with the p210 isoform appeared to show a trend toward improved OS and RFS compared with those with the p190 isoform (Figures 2D,E).

### Safety

**Figure 3:**
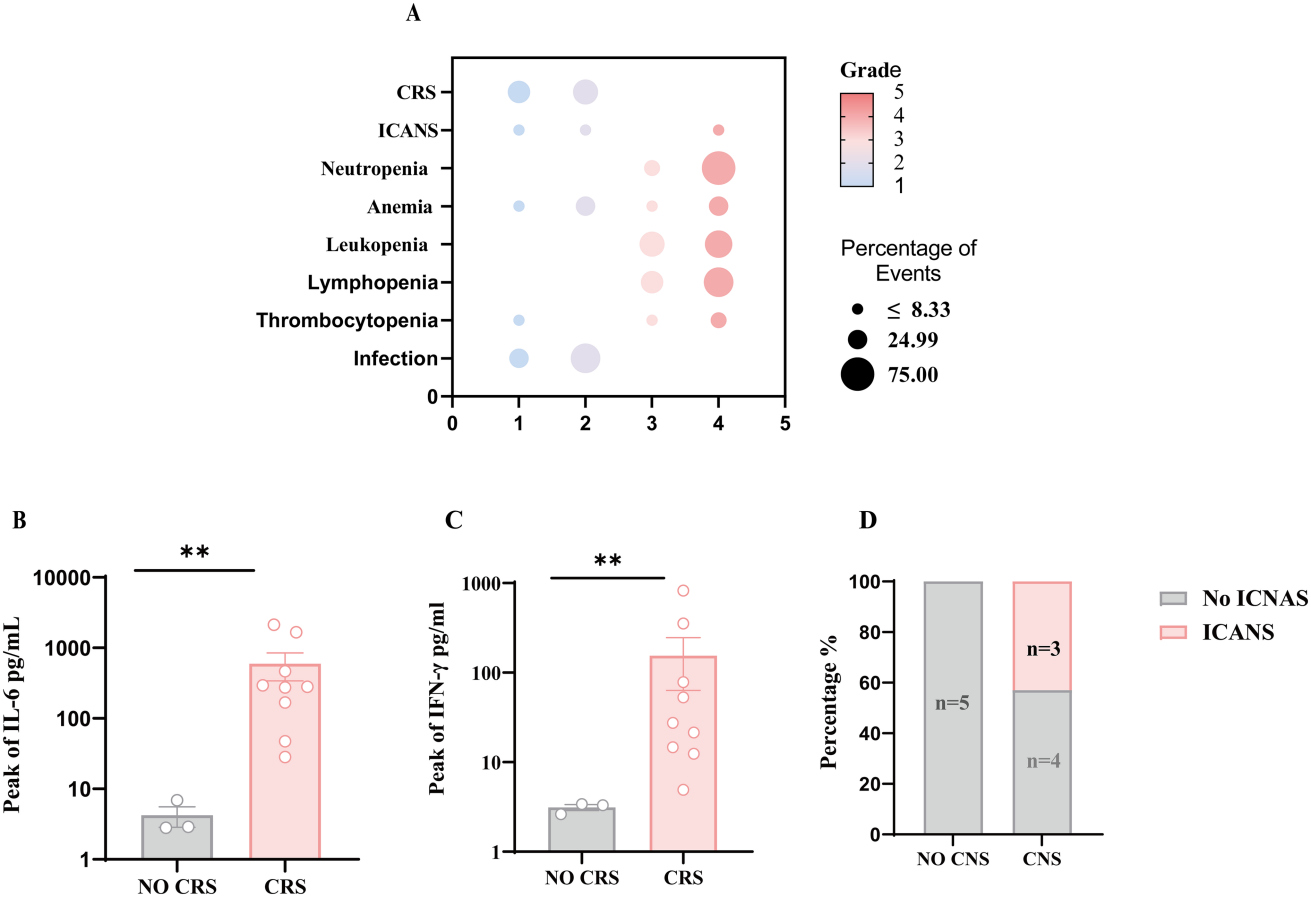
Safety characteristics of CD19 and CD22 CAR-T cells infusion. **(A)** Bubble plots representing the frequency of the indicated adverse events for 12 Ph^+^ ALL patients. The color spectrum corresponds to adverse event grades 1–5; bubble size corresponds to frequencies. **(B,C)** Comparison of the median peak of IL-6 and IFN-γ between patients with (*n* = 9) and without CRS (*n* = 3), using the Mann–Whitney *U* test. **(D)** Number of patients with ICANS (*n* = 3) according to whether or not they have CNS involvement. ** *p* <0.01 indicate levels of statistical significance.

During treatment, CRS occurred in 9 (85.7%) patients, including 7 cases of grade 1 and 2 cases of grade 2 (Figure 3A). Notably, patients with CRS had a significantly increased median peak of IL-6 and IFN-γ level compared with those without CRS (2.9 vs. 281.8 pg./mL; 3.3 vs. 27.44 pg./mL; *p* = 0.0091) (Figures 3B,C). Tocilizumab (5, 41.7%) and/or corticosteroids (3, 25%) were used according to symptomatic supportive management; all symptoms of CRS demonstrated reversibility. Three (25%) patients experienced ICANS, which was grade ≥3 in 1 patient, and all occurred in patients with isolated or combined CNS relapse (Figure 3D). None of these patients experienced seizures or loss of consciousness. Ten (83.3%) patients had infections after CAR-T cell infusion, and all were grade 1 and 2. In addition, Cytopenia was the most common adverse event and was all manageable without treatment discontinuation. These included neutropenia (91.7%), leukopenia (91.7%), anemia (66.7%), and thrombocytopenia (33.3%). Significantly, no patients died due to severe adverse events. Detailed AEs are presented in Table 2.

**Table 2:** Safety outcomes among all enrolled patients.

Adverse events	N/ratio	Grade ≥ 3
Fever	9 (75%)	0
CRS	9 (75%)	0
ICANS	3 (25%)	1 (8.3%)
Hypotension	1 (8.3%)	0
Hypoxemia	1 (8.3%)	0
Neutropenia	11 (91.7%)	11 (91.7%)
Anemia	8 (66.7%)	4 (33.3%)
Leukopenia	11 (91.7%)	11 (91.7%)
Lymphopenia	11 (91.7%)	10 (83.3%)
Thrombocytopenia	4 (33.3%)	3 (25%)
Infection	10 (83.3%)	0
Received tocilizumab	5 (41.7%)	/
Received corticosteroids	3 (25%)	/

CRS, cytokine release syndrome; ICANS, immune effector cell-associated neurotoxicity syndrome; /, None.

### CAR-T cell expansion and persistence

**Figure 4:**
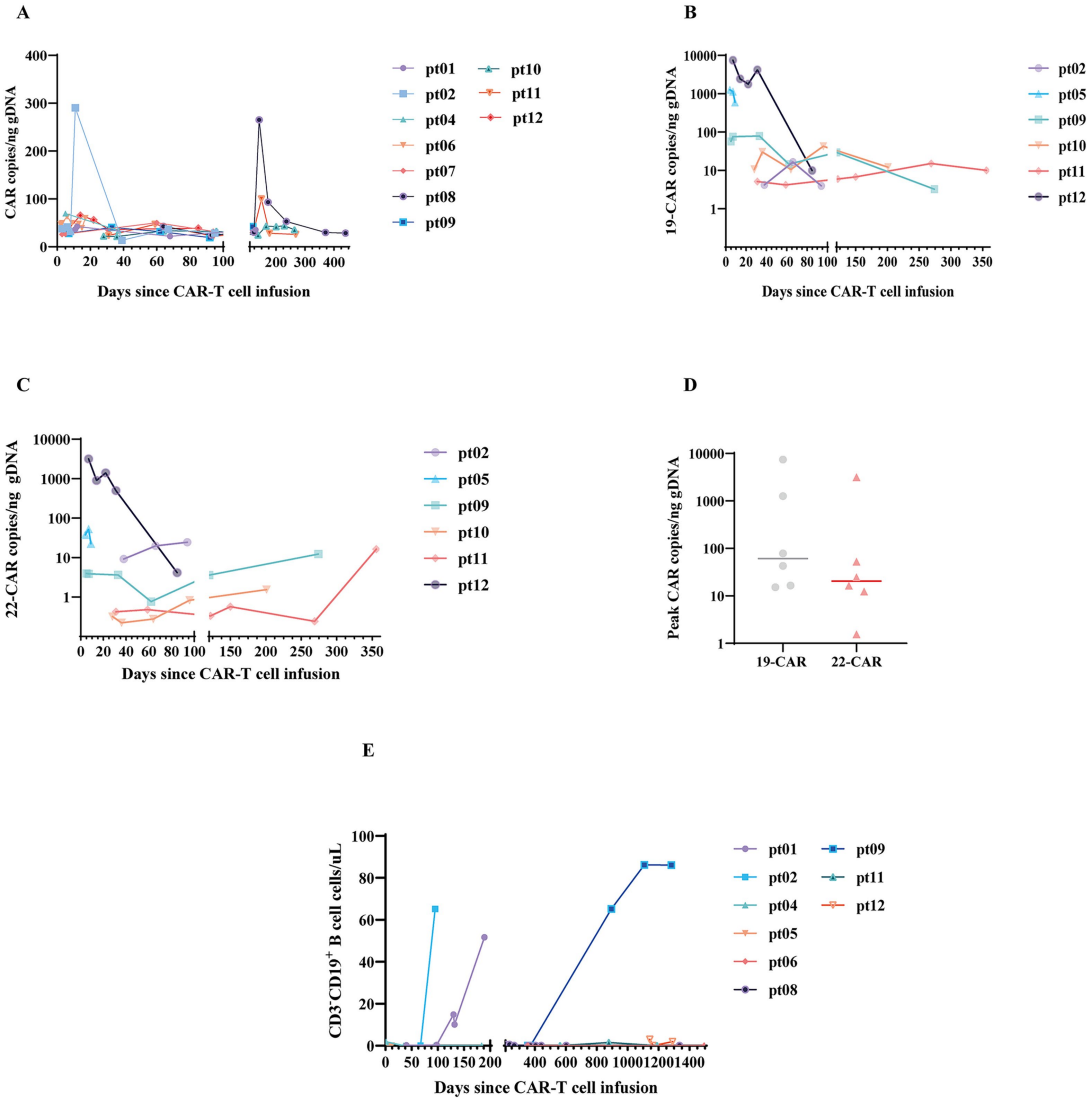
Expansion and persistence of CD19 and CD22 CAR-T cells. **(A)** Total CAR-T cells in the peripheral blood (PB) of 10 patients as detected using quantitative real-time PCR. **(B)** Kinetics of CD19-CAR-T cells in the PB of 6 patients. **(C)** Kinetics of CD22 CAR-T cells in the PB of 6 patients. **(D)** Dot plot illustrating peak CD19 CAR-T cells and CD22 CAR-T cells expansion in 6 patients. Each data point represents an individual patient, and horizontal lines denote the median value. **(E)** Kinetics of B cells in PB from 9 patients, determined by FC.

Of 12 patients, 10 with available data in PB (Figure 4A) had significant CAR-T cell expansion after CAR-T cell infusion, with a median C_max_ of 59.56 CAR copies/ng gDNA (range, 42.04–290.2 copies/ng gDNA). Besides, among 6 of these 10 patients, the median peak copies of CD19 CAR-T cells were higher than those of CD22 CAR-T cells (60.86 vs. 20.5 copies/ng gDNA) (Figures 4B–D). CAR-T cell persistence examination by FCM or qPCR was observed for more than 6 months in five patients, with two patients (Pt05, Pt06) still having detectable CAR-T cells beyond 24 months.

BCA was detected in peripheral blood in 11 patients, and the median duration of BCA was 11.8 months (range, 1.4–44.5 months). Four patients still exhibited BCA by 30 months post-infusion, and all of them remained in sustained CHR (Figure 4E).

## Discussion

Chemotherapy-free regimens based on TKIs and immunotherapy have substantially improved survival outcomes in Ph^+^ ALL (15). However, in pediatric patients with R/R Ph^+^ ALL, the long-term outcomes of CD19 and CD22 dual-target CAR-T therapy remain incompletely characterized. In this retrospective subanalysis, co-administration of CD19 and CD22 CAR-T cell therapy achieved 100% CHR and MRD^−^CR, with a CMR rate of 77.8% among evaluable patients and encouraging long-term survival, including a 4-year OS of 91.7% and a 4-year RFS of 66.7%. These preliminary findings may provide insight into the potential role of dual-targeted CAR-T therapy in children with R/R Ph^+^ ALL.

Previous studies have shown that dual-targeted CAR-T strategies, including bicistronic or tandem dual CARs, coadministration, and sequential CAR-T cell infusion approaches, may offer advantages over single-target CAR-T therapy in patients with R/R B-ALL (17, 19–21). Our findings are consistent with these observations, showing efficacy comparable to the 100% MRD response reported in a phase I study of CD19/CD22 bispecific CAR-T cells that included seven patients with Ph^+^ ALL (22), and superior to the 84.62% MRD-CR and 53.85% CMR rates reported with CD19 CAR-T therapy in pediatric R/R Ph^+^ ALL (23). In addition, the combination of ponatinib and blinatumomab has shown promising efficacy in R/R Ph^+^ ALL. Jabbour et al. reported an overall remission rate (ORR) of 92% and a CMR rate of 79% in 14 patients (24). Nonetheless, the efficacy of blinatumomab monotherapy appears limited, as evidenced by the ALCANTARA phase II trial, in which 31% of 45 patients achieved complete remission (CR) after one cycle, with median RFS and OS of 6.8 and 9.0 months (25). Within the context of these previous studies, the long-term outcomes observed in our cohort appear encouraging, with a 4-year OS of 91.7% and a 4-year RFS of 66.7%.

Mechanistically, current evidence suggests that dual-target CAR-T therapy may reduce selective pressure on a single antigen, thereby reducing the risk of antigen-loss-driven relapse (26, 27). In a previous trial of 26 patients who relapsed after tandem CD19/CD20 CAR-T cell infusion, only one patient developed CD19/CD20 double-negative relapse (28). Similarly, among the four patients who relapsed in our cohort, none developed CD19-negative relapse.

The molecular and genomic heterogeneity of Ph^+^ ALL contributes to substantial variability in clinical outcomes and has important implications for treatment optimization (29). Although p190-positive ALL has historically been associated with a more favorable outcome (30), our exploratory analysis suggested a trend toward improved OS and RFS in patients with the p210 isoform; however, this observation should be interpreted cautiously given the small sample size. Moreover, adverse genetic features are associated with inferior outcomes (31). In our cohort, five patients harbored genetic alterations, including ABL1 kinase domain mutations, IKZF1 deletion/IK6 abnormality and additional mutations involving epigenetic regulators such as WHSC1, KMT2D, and BCORL1. Notably, IKZF1 deletion has been associated with inferior OS and an increased risk of relapse in patients with Ph^+^ ALL (32). Consistent with this observation, two of the three patients in our cohort with IKZF1 deletion experienced relapse, and one died during follow-up. In contrast, one patient with ABL1 kinase domain mutations and extensive prior TKI exposure achieved long-term survival after CAR-T therapy followed by bridging allo-HSCT and ponatinib-based treatment, suggesting that an individualized strategy may be more effective in select high-risk cases.

CNS involvement remains a critical concern in B-ALL, particularly given the elevated risk associated with Ph^+^ ALL. Tan et al. retrospectively analyzed the efficacy of CD19 CAR-T cell therapy in 12 pediatric patients with refractory high-burden CNSL (defined as blasts >20/μL in CSF or intracranial mass lesions), among whom 11 patients (91.7%) achieved complete remission within 30 days, and the 6-month LFS rate was 81.8% (33). Furthermore, in a multicenter retrospective study of CD19-specific CAR T-cell therapy for R/R B-ALL with CNSL (confirmed as CNS-3 status at the time of the most recent relapse or within 30 days before screening), Qi et al. showed that patients with Ph^+^ ALL had significantly inferior event-free survival and OS compared with those without *BCR::ABL1* rearrangement (12). In this context, the outcomes observed in our cohort are encouraging. Among the seven patients in our cohort with CNS relapse, the 4-year OS and RFS rates were 100 and 71.4%. Notably, only one of the four patients with CNS-3 status developed subsequent CNS relapse.

Historically, allo-HSCT has been widely regarded as a potentially curative intervention for Ph^+^ ALL, with achievement of complete remission at the time of transplantation serving as a critical prerequisite for favorable outcomes (34–36). However, its application is significantly constrained by donor availability and associated transplant-related complications. In a retrospective analysis of 267 patients with Ph^+^ ALL, 5-year OS rates were 44% in the sibling allo-HSCT group, and 36% in the matched unrelated donor (MUD) allo-HSCT group, whereas treatment-related mortality (TRM) was notable, with 27% in the sibling allo-HSCT group and 39% in the MUD allo-HSCT group (37).

Moreover, with the advent of immunotherapy, the role of consolidative allo-HSCT after remission has become less clear in certain settings. In a multicenter retrospective study including 93 patients with R/R Ph^+^ ALL treated with CD19 CAR-T cell, consolidative allo-HSCT was not independently associated with improved OS or LFS (38). Similarly, a prospective study of CD19 CAR-T therapy reported that two patients maintained durable remission without subsequent allo-HSCT (39).

Additional evidence from dual-targeted CAR-T cell therapy is consistent with this evolving paradigm. In a phase I study of CD19/CD22 bispecific CAR-T cells in MRD-positive ALL, nine patients, including five patients with Ph^+^ ALL, achieved sustained remission without subsequent allo-HSCT until the end of follow-up (22). Likewise, in a frontline study combining dasatinib with sequential CD19/CD22 CAR-T therapy, the CMR rate and 2-year LFS rate were 85 and 92%, with only one patient undergoing consolidative allo-HSCT (40).

In our cohort, six patients achieved sustained hematologic remission without consolidative allo-HSCT. Following TKI treatment, two of these patients (Pt09, Pt10) with *BCR::ABL1-*positive CHR regained CMR. Interestingly, only one patient (Pt08) continued dasatinib maintenance while in CMR. Notably, we also observed that two patients (Pt03, Pt12) who received consolidative allo-HSCT maintained CMR and were alive at 60 months of follow-up, suggesting that both strategies may yield durable outcomes in select cases.

Taken together, CAR-T cell therapy may offer an alternative to consolidative allo-HSCT in select cases, and further prospective studies are warranted to define optimal patient selection.

## Conclusions and limitations

In conclusion, these preliminary findings suggest CD19 and CD22 CAR-T cell therapy may provide long-term survival benefits in pediatric patients with R/R Ph^+^ ALL.

However, several limitations should be acknowledged. First, the small sample size limits the generalizability of our findings and precludes robust statistical analysis. Second, the retrospective selection of the study cohort, incomplete data, and follow-up information partially obtained from family reports may have introduced bias. Finally, patient heterogeneity and the absence of a control group limit the ability to definitively attribute the observed outcomes to CD19/CD22 CAR-T therapy.

Consequently, these findings are preliminary and hypothesis-generating, warranting validation in larger, prospective, controlled trials with longer follow-up to further evaluate its potential value in patients with Ph^+^ ALL.

## Data Availability

The raw data supporting the conclusions of this article will be made available by the authors, without undue reservation.
