# Supplementary material for: Long-term outcomes of co-administration of CD19 and CD22 CAR-T cell therapy in pediatric patients with relapsed/refractory Philadelphia chromosome-positive acute lymphoblastic leukemia
Source: Front Med (Lausanne). 2026 Apr 10;13:1815353. doi: 10.3389/fmed.2026.1815353 (PMC13105937; doi:10.3389/fmed.2026.1815353)
Supplement: Supplementary file 1 [file Supplementary_file_1.docx]

**Supplementary materials**

**Supplementary Figure Legends**

**
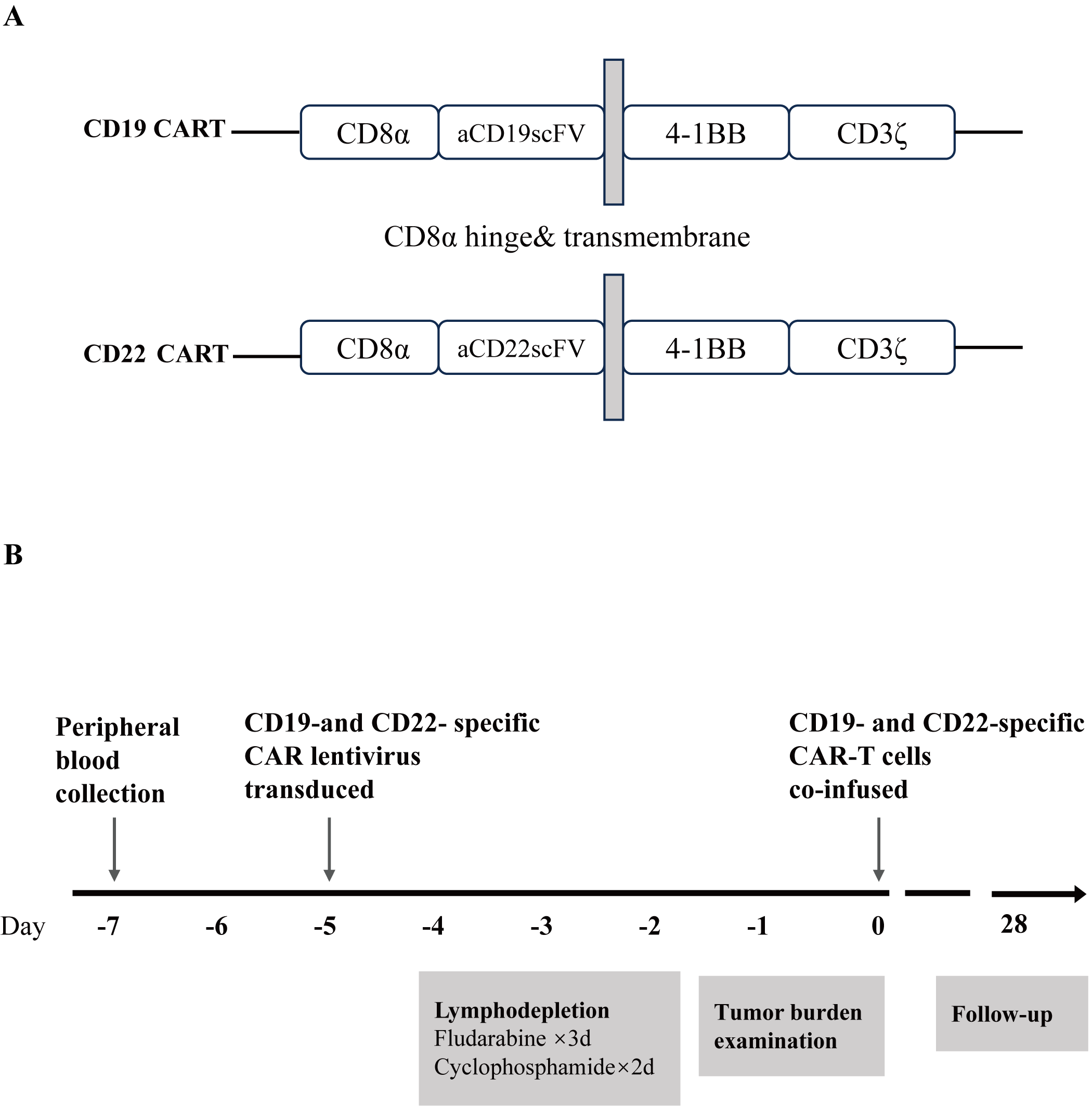
**

**Supplementary Figure 1:** **Schematic of the CD19-CAR and CD22-CAR constructs and the workflow of CAR-T cell therapy.**

**A,** Schematic illustration of CD19-CAR and CD22-CAR construct; **B,** Flowchart of CD19 and CD22 CAR-T cell manufacturing and treatment process.

| **Supplementary table 1: CD19 and CD22 CAR-T products' characteristics** | | | | | | | | |
| --- | --- | --- | --- | --- | --- | --- | --- | --- |
| **Patient ID** | **culture cycle (day)** | **Fresh/**  **Cryopreserved** | **Weight (kg)** | **Transduction Efficiencies of CD19-CAR** | **Transduction Efficiencies of CD22-CAR** | **Total CAR-T dose ×10^6^/kg** | **CD19- CAR dose ×10^6^/kg** | **CD22-CAR dose ×10^6^/kg** |
| **pt01** | 7 | Fresh | 22.0 | 74.0% | 77.4% | 5.1 | 2.5 | 2.6 |
| **pt02** | 10 | Fresh | 38.1 | 41.1% | 39.5% | 6.7 | 3.4 | 3.2 |
| **pt03** | 7 | Fresh | 17.0 | 44.7% | 37.3% | 9.7 | 5.6 | 4.1 |
| **pt04** | 7 | Fresh | 22.4 | 52.4% | 67.2% | 4.0 | 1.9 | 2.1 |
| **pt05** | 7 | Fresh | 53.0 | 28.8% | 33.3% | 3.8 | 1.8 | 2.0 |
| **pt06** | 9 | Fresh | 60.0 | 43.5% | 34.1% | 3.9 | 2.2 | 1.7 |
| **pt07** | 8 | Fresh | 90.0 | 36.0% | 24.9% | 3.0 | 1.8 | 1.2 |
| **pt08** | 7 | Fresh | 17.0 | 64.5% | 68.9% | 3.3 | 1.6 | 1.7 |
| **pt09** | 8 | Fresh | 57.0 | 73.4% | 65.8% | 6.0 | 3.2 | 2.9 |
| **pt10** | 10 | Fresh | 33.0 | 26.8% | 49.8% | 2.3 | 0.8 | 1.5 |
| **pt11** | 10 | Fresh | 30.0 | 40.7% | 52.8% | 11.2 | 4.9 | 6.3 |
| **pt12** | 7 | Fresh | 25.0 | 57.1% | 63.9% | 7.5 | 2.5 | 2.4 |

| **Supplementary table 2: Clinical timelines for patients** | | | | | | |
| --- | --- | --- | --- | --- | --- | --- |
| **Patient ID** | **Diagnosis** | **Diagnosis date** | **Chemotherapy regimens** | **Relapse/Refractory date** | **Enrollment Criteria** | **CAR-T infusion  date** |
| **pt01** | ^#^Ph^+^ ALL | 2019.05.03 | *CCCG-ALL-2015 | 2021.04.20 | Relapse | 2021.06.03 |
| **pt02** | Ph^+^ ALL | 2018.08.06 | CCCG-ALL-2015 | 2021.07.29 | Relapse | 2021.08.15 |
| **pt03** | Ph^+^ ALL | 2018.10.09 | CCCG-ALL-2015 | 2019.10.01 | Refractory | 2019.11.14 |
| **pt04** | Ph^+^ ALL | 2017.10.15 | CCCG-ALL-2015 | 2021.05.31 | Relapse | 2021.06.15 |
| **pt05** | Ph^+^ ALL | 2018.05.16 | CCCG-ALL-2015 | 2021.08.24 | Relapse | 2021.09.29 |
| **pt06** | Ph^+^ ALL | 2017.06.04 | CCCG-ALL-2015 | 2019.12.01 | Relapse | 2020.11.28 |
| **pt07** | Ph^+^ ALL | 2017.03.20 | CCCG-ALL-2015 | 2020.09.28 | Relapse | 2020.11.22 |
| **pt08** | Ph^+^ ALL | 2015.10.09 | CCCG-ALL-2015 | 2021.05.21 | Relapse | 2021.06.16 |
| **pt09** | Ph^+^ALL | 2018.02.02 | CCCG-ALL-2015 | 2021.01.08 | Relapse | 2021.01.20 |
| **pt10** | Ph^+^ ALL | 2017.08.02 | CCCG-ALL-2015 | 2020.05.12 | Relapse | 2020.12.27 |
| **pt11** | Ph^+^ ALL | 2018.01.22 | CCCG-ALL-2015 | 2020.09.25 | Relapse | 2020.10.27 |
| **pt12** | Ph^+^ ALL | 2015.05.22 | CCCG-ALL-2015 | 2019.12.17 | Relapse | 2020.10.07 |
| ^#^Ph^+^ ALL: Philadelphia chromosome-positive Acute Lymphoblastic Leukemia; *CCCG-ALL-2015: the Chinese Children's Cancer Group ALL-2015 protocol. | | | | | | |
